# Simplified Automated Image Analysis for Detection and Phenotyping of *Mycobacterium tuberculosis* on Porous Supports by Monitoring Growing Microcolonies

**DOI:** 10.1371/journal.pone.0011008

**Published:** 2010-06-08

**Authors:** Alice L. den Hertog, Dennis W. Visser, Colin J. Ingham, Frank H. A. G. Fey, Paul R. Klatser, Richard M. Anthony

**Affiliations:** 1 KIT Biomedical Research, Royal Tropical Institute, Amsterdam, The Netherlands; 2 Vleuten, The Netherlands; 3 Microdish BV, Houten, The Netherlands; 4 CCM Centre for Concepts in Mechatronics, Nuenen, The Netherlands; University of Delhi, India

## Abstract

**Background:**

Even with the advent of nucleic acid (NA) amplification technologies the culture of mycobacteria for diagnostic and other applications remains of critical importance. Notably microscopic observed drug susceptibility testing (MODS), as opposed to traditional culture on solid media or automated liquid culture, has shown potential to both speed up and increase the provision of mycobacterial culture in high burden settings.

**Methods:**

Here we explore the growth of *Mycobacterial tuberculosis* microcolonies, imaged by automated digital microscopy, cultured on a porous aluminium oxide (PAO) supports. Repeated imaging during colony growth greatly simplifies “computer vision” and presumptive identification of microcolonies was achieved here using existing publically available algorithms. Our system thus allows the growth of individual microcolonies to be monitored and critically, also to change the media during the growth phase without disrupting the microcolonies. Transfer of identified microcolonies onto selective media allowed us, within 1-2 bacterial generations, to rapidly detect the drug susceptibility of individual microcolonies, eliminating the need for time consuming subculturing or the inoculation of multiple parallel cultures.

**Significance:**

Monitoring the phenotype of individual microcolonies as they grow has immense potential for research, screening, and ultimately *M. tuberculosis* diagnostic applications. The method described is particularly appealing with respect to speed and automation.

## Introduction

Mycobacterial diagnostic provision and research is labour intensive, potentially dangerous and slow. Automation of microbiological techniques has lagged behind other disciplines. Reducing the time to detection of bacterial growth is important for diagnostic microbiology and a critical issue for the detection of slow growing organisms such as *Mycobacterium tuberculosis* (MTB). Traditional culture methods take a number of weeks to complete a diagnosis and even “automated” methods require considerable hands on time. With the emergence of drug resistant strains, and drug susceptibility testing thus becoming increasingly important, the delay caused by culturing is even more significant.

The use of automated liquid culture methods, already widely used in industrialized countries, has recently been recommended for consideration in many more countries to improve time to detection [1], [2]. However, liquid culture, although effective is not ideal, requiring highly skilled microbiologists to perform extensive post-culture identification strategies and ensure biological safety [3].

For this reason there is considerable interest in the microscopic detection of early mycobacterial colonies, to date most often performed in liquid media by the “microscopic observation of broth cultures” (MODS) method [4]. This method results in a reduced time to detection, and may be more practical in high burden countries. MODS as currently implemented is labour intensive, because microcolonies suspended in liquid media must be visually examined at multiple time points. For susceptibility testing using the MODS method, samples are inoculated in parallel in wells containing media with and without antibiotics so culture and DST are performed in parallel in one assay [4]–[6]. Colony growth is visually analysed with an inverted microscope. The distinctive morphology of floating microcolonies allows trained human observers to readily identify them, but complex (3D) shape recognition tasks are notoriously difficult to automate with a high degree of sensitivity and specificity [7],[8]. Furthermore, an identified floating microcolony cannot be tracked effectively over time.

Thin layer agar (TLA) culture, which is also being explored as an alternative to automated liquid culture in high burden countries, is similar to MODS in that cultures are examined for microcolony growth by microscopic analysis, but is performed on solid medium [9].

Here we demonstrate an alternative approach for microcolony detection specifically conceived to be easy to automate. This method combines automated colony detection with the possibility to perform rapid DST on individually formed colonies. Individual microcolonies are detected and monitored over time allowing presumptive identification based on their growth.

Specifically, the microcolonies are grown on porous aluminium oxide supports, which are highly porous, inert and optically flat solid membranes that can be used in combination with any (solid or liquid) media [10], [11].

Growing on solid porous supports has the additional advantage that established microcolonies can be transferred to different culture medium, while growth is monitored before and after transfer. Thus, effects of altered culture conditions can be measured for each colony, allowing for individual colony based DST and thus identification of mixed phenotypes.

This possibility to transfer many colonies simultaneously to selective media is analogous to the “replica plating” using felt pads, which although a very powerful technique, is not practical for diagnostic purposes [12]. Instead, for sequential diagnostic susceptibility testing (or other post-culture analysis) generally only very few “representative” colonies are selected for further testing.

With the method presented here, the response of each colony to altered (selective) medium can be monitored while the lag phase introduced by sub-culturing individual cells is eliminated. We believe this approach is particularly amenable to automation, can be implemented in such a way as to minimize infection risk and provides many opportunities to investigate bacterial response to antibiotics that would be impractical with other methods.

## Results

### Real-time microcolony growth microscopy

#### Effect of PAO on growth rate

**Figure 1:**
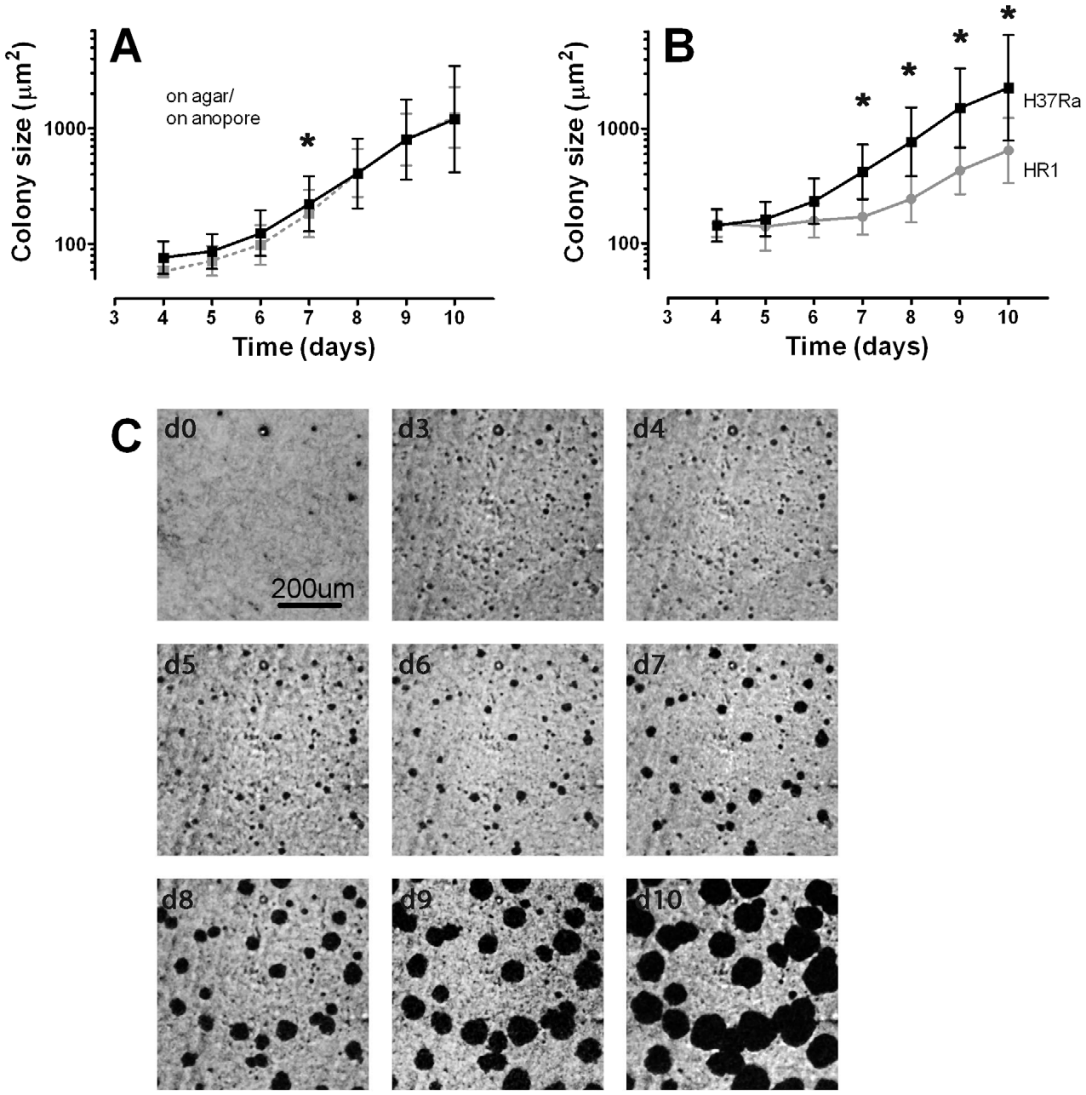
Growth of microcolonies on PAO. A: Growth curves of H37Ra on PAO on agar (continuous black line), or directly on agar (dashed grey line). B: Growth curves of H37Ra (▪) and HR1 (•) on PAO on agar. Average colony size is plotted; bars indicate ±1 standard deviation of measured colony sizes. Statistically significant differences between (A) PAO and agar or (B) H37Ra and HR1 (tested at days 7–10) are indicated by an asterisk (*; p<0.001).C: Example images of a single microscope field (705 µm×705 µm) taken at inoculation (d0) and daily between 3 and 10 days after inoculation.

To measure any effect of the PAO support on the growth rate of the colonies, we monitored the growth rate of bacteria inoculated in parallel on agar and on PAO (Figure 1A). Measured colony size of H37Ra was slightly higher on PAO compared to agar until day 7, thereafter the growth on PAO and on agar was indistinguishable.

#### Initial definition of growth characteristics

In order to define the growth characteristics of MTB on PAO, a number of *M. tuberculosis* strains were inoculated on PAO and their growth monitored daily for 11 days. These data were used to set a window of expected daily growth rate of MTB colonies for automated analysis (between 25% to 200% increase in area over 24 hours). As would be expected not all isolates grew at exactly the same rate. For example the RIF resistant strain HR1 (H526Y) had a decreased growth rate compared to its parent strain H37Ra (Figure 1B) but still falls within the defined window.

#### Automated analysis

In this study the smallest objects in the microscopic images considered to be potentially colonies had an area of between 50 and 100 pixels (178–356 µm^2^). Colonies within this size range started to become visible within 4 days (Figure 1C) but could not be definitively identified as colonies at this point as other structures in this size range were also present for example debris, air bubbles and scratches. Automated monitoring of the increase in size of any objects present at day 4 or later was used to identity the true colonies. Thus, we included only objects present in at least two subsequent time points that increased in size within the expected range (25–200%) in our analysis.

### High power microscopy of inocula and microcolonies

In order to validate our method high power microscopy of fixed and stained cells/microcolonies was used to give an indication of the numbers of colonies formed by more than one bacterial cell inoculated and present in the smallest microcolonies observable at the lower magnification.

First the inoculum was examined at high power after auramine staining of glass slides containing inoculation suspension of H37Ra. The inoculum was seen to consist of >80% single cells.

**Figure 2:**
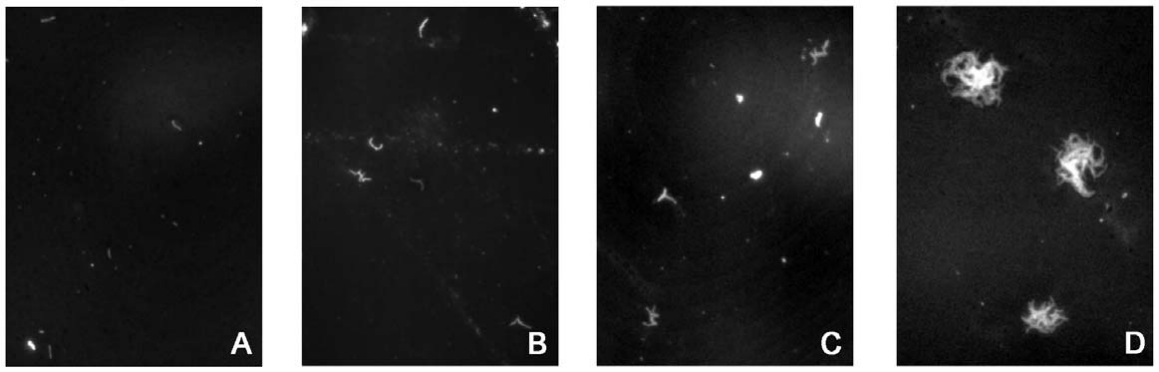
Colony formation on PAO. A–D; H37Ra cultured for 1, 2, 3 and 7 days on PAO were fixed and stained with Nile Red and examined with a 40X objective using fluorescent epi-illumination. Representative microcolonies for each time point are shown.

Subsequently, Nile Red staining of PAO filters inoculated with H37Ra and fixed on day 0 was used to confirm that the vast majority of bacteria present on the PAO strips remained as single cells, although occasional paired cells were also seen. After 24 hours incubation most cells were elongated and some small clusters of cells (2–3 cells) were seen after Nile Red staining of bacteria on PAO, after 2 days most microcolonies consisted of 4–8 cells (Figure 2A,B). After 3 days, most colonies consisted of between 5–15 cells and at day 7 most microcolonies consisted of 50–200 cells (Figure 2C,D).

### Colony growth after incubation on selective media

To determine colony growth on PAO in the presence of selective agents, paired rifampicin resistant and susceptible *M. tuberculosis* strains were inoculated on PAO in duplicate on non-selective medium as described above, and images were recorded at the time of inoculation and days 3 to 7. At day 7, the PAO strips of both the susceptible and resistant strains were moved onto fresh agar with and without 8 µg/ml rifampicin and growth measurements continued up to day 10.

**Figure 3:**
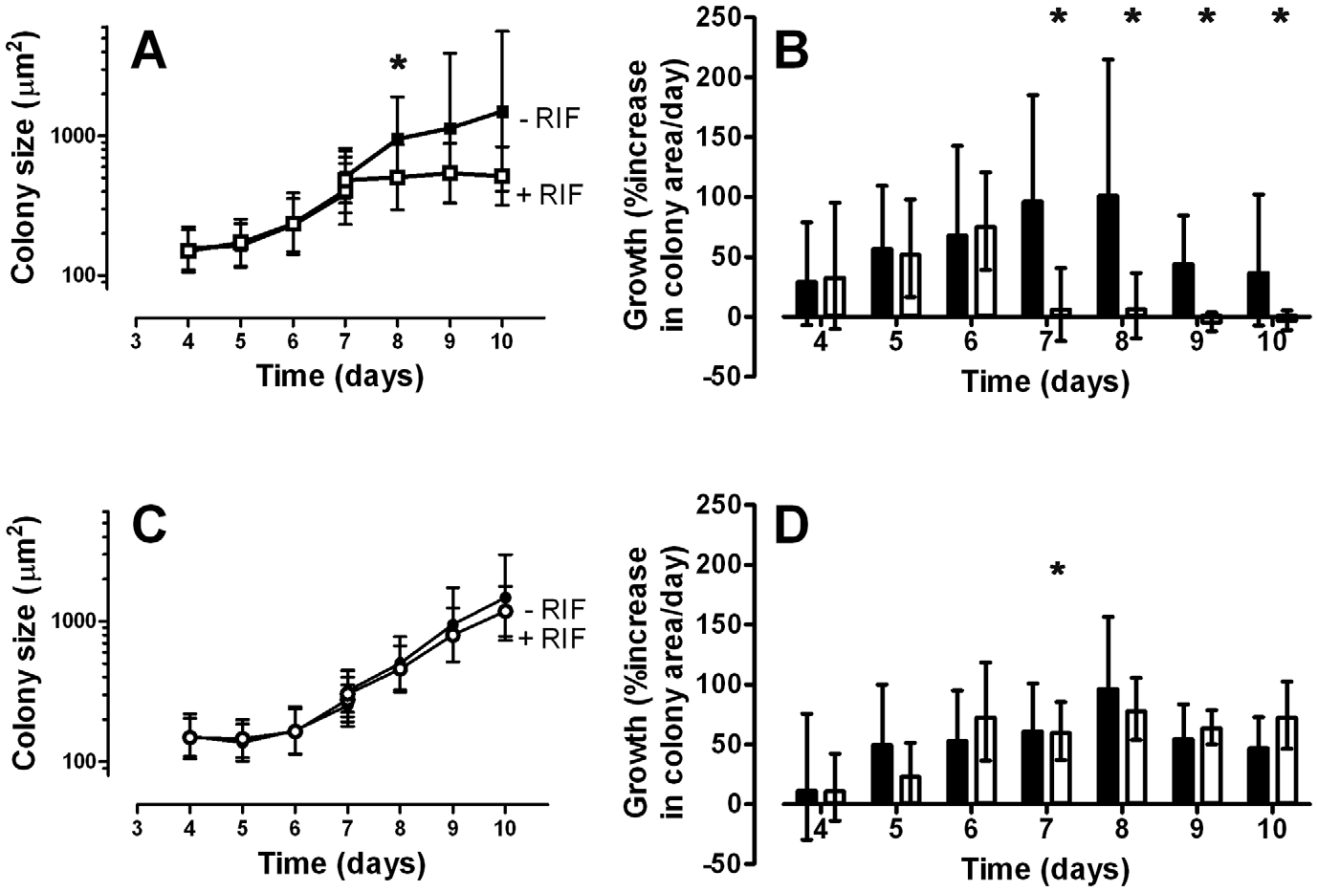
Growth of strains H37Ra and HR1 in the presence of RIF. Microcolonies of RIF susceptible strain H37Ra and resistant strain HR1 were grown without RIF for 7 days, then moved to fresh agar with or without RIF from day 7 onwards. A, C: Averaged sizes of H37Ra (A) and HR1 (C) colonies incubated with (open symbols) and without RIF (closed symbols). Average colony size is plotted; bars indicate ±1 standard deviation of measured colony sizes. A statistically significant difference (indicated by an asterisk (*), p<0.001) between the groups at day 7 and 8 (unpaired T-test) was found between H37Ra with and without RIF at day 8. B: Growth rate of individual H37Ra (B) and HR1 (D) colonies incubated with (white bars) and without (black bars) RIF. Statistical analysis was performed using a paired T-test on colony growth between day 6 and day 7, and day 6 and day 8. Statistical significance is indicated by an asterisk (*; p<0.001). Although there is a statistically significant difference between the growth rate of HR1 at day 6 and 7 in the presence of RIF, the change in growth rate is so small compared to the sensitive strain that we consider this effect irrelevant.

At day 8, at 24 h after transfer, a difference could be seen between the average sizes of the colonies of the susceptible strain with and without RIF (Figure 3A), whereas no such difference could be seen in the resistant strain (Figure 3C). The effect on the susceptible strain was even more apparent when the growth rate of individual colonies was measured, in fact the growth effectively stopped within the first 24 h after addition of the antibiotic (Figure 3B,D). Similar data were obtained with other paired resistant and susceptible strains (data not shown).

High power microscopy of the PAO strips fixed and stained with Nile Red after ending the experiment (at day 11) confirmed that the colonies of the susceptible strains incubated with RIF where indeed much smaller than the controls (data not shown).

### Detection of mixed strains incubated on selective media

To study the impact of mixed cultures on this analysis strategy, we also inoculated PAO strips with mixtures of the RIF susceptible and resistant strains. The experimental procedure used was identical to that used above; after 7 days of culture, paired samples were placed on media with or without RIF.

**Figure 4:**
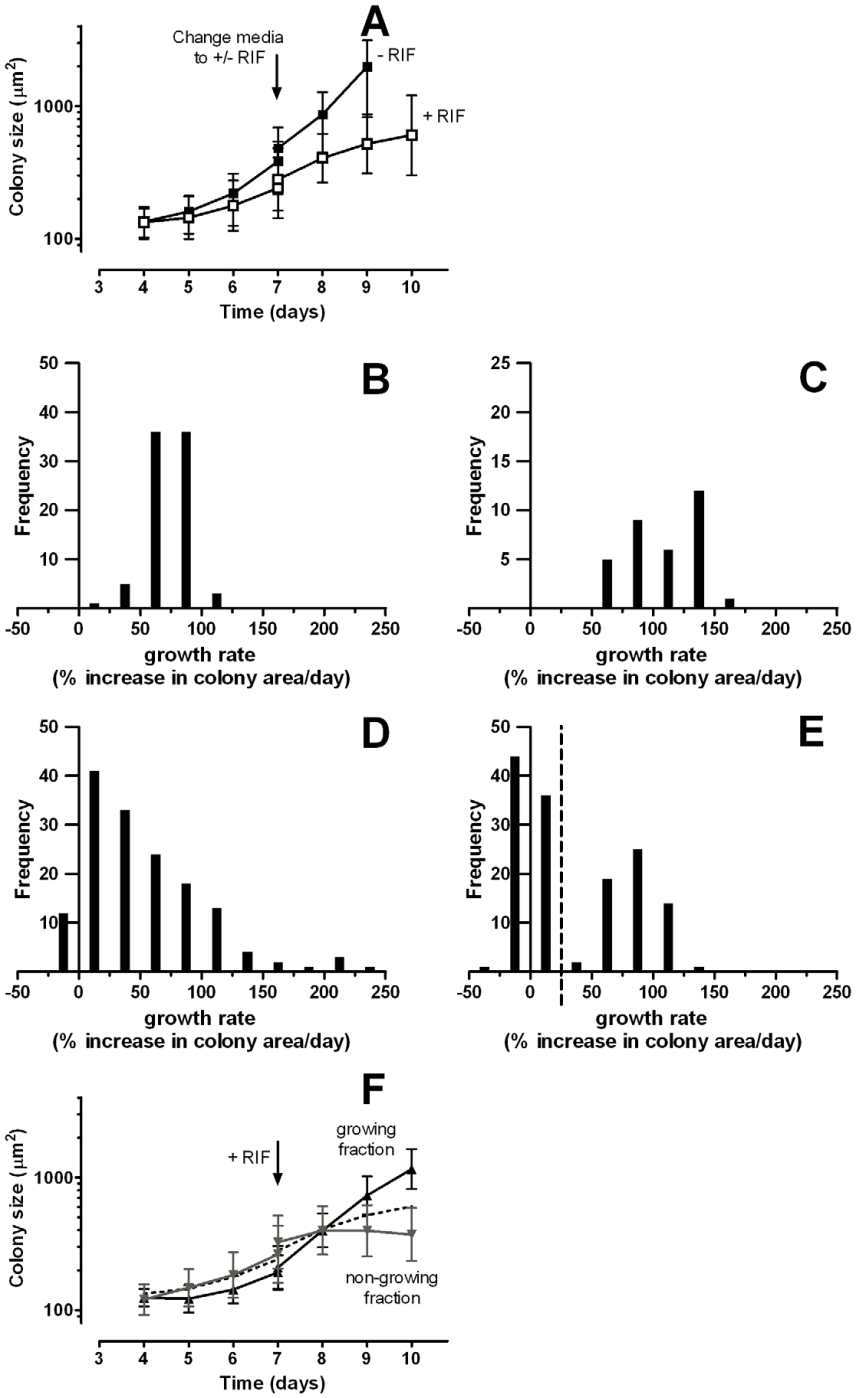
Growth of mixed strains H37Ra and HR1 in the presence of RIF. Microcolonies of mixed cultures of H37Ra and HR1 were grown without RIF for 7 days, then moved to fresh agar with or without RIF from day 7 onwards. A: Average colony sizes of total mixed population incubated with (▪) and without (□) RIF. Average colony size is plotted; bars indicate ±1 standard deviation of measured colony sizes. B, C: Distribution of growth rates (% increase/day) at day 7 (B) and 8 (C) of the total mixed population without RIF exposure. D, E: Distribution of growth rates (% increase/day) at day 7 (D) and 8 (E) of the total mixed population after RIF exposure. In E the vertical dashed line indicates the cut-off value (25%) for growth defined on basis of measured growth rate at day 8. F: Averaged colony sizes of the mixed population with RIF separated into the growing (RIF resistant, black line) and non-growing (RIF susceptible, grey line) populations on basis of the cut-off value shown in E. Average colony size is plotted; bars indicate ±1 standard deviation of measured colony sizes. For reference, data for the complete mixed population is indicated by the dashed line.

Even with a mixed culture, when analysing the whole population as one, in the RIF treated population an immediate decrease in the growth rate was seen when compared to the non-RIF treated population (Figure 4A), although this decrease was not as dramatic as in the pure RIF susceptible population (Figure 3A). On an individual colony basis the growth rate distribution of the non-RIF treated sample gave a single peak at all time points (Figure 4B,C). For the mixed culture on RIF at day 7 the range of growth rates was already wider than in the absence of RIF (Figure 4D). At day 8 two populations could be distinguished (Figure 4E). Therefore, on basis of the growth rates of individual colonies at day 8 it was possible to retrospectively classify individual colonies as growing or non-growing (using a cut-off value of 25% increase in colony size per day; indicated in Figure 4E). Thus on basis of the growth rates we assigned each colony to the resistant or susceptible population and average growth curves were plotted for each population (Figure 4F), clearly showing 2 discrete populations.

## Discussion

Measuring at multiple time points greatly simplifies automated microcolony detection and identification. When colonies first become detectable is inevitably very difficult from a single image to unequivocally identify them as such, either electronically or by eye. By imaging at sequential time points and calculating the rate of change in object size, colonies with growth characteristics consistent with an *M. tuberculosis* colony can be identified. Furthermore, by continuing to monitor identified microcolonies after transferring the PAO support to selective media, the response of individual colonies can be rapidly and accurately determined. The recent rapid development in CCD camera technology, LED illumination and massive reduction in the cost of computer processing power and storage make this type of approach increasing accessible but automated detection of objects in images remains challenging [7], [8]. Here we present a strategy specifically designed to generate data that is particularly suited to automated analysis and show this can be achieved using a existing image analysis tools and a dedicated automated microscope.

Initially, we demonstrated the PAO does not restrict access to nutrients as *M. tuberculosis* colonies grown on PAO supports on agar have equivalent growth rates to colonies grown directly on agar. Monitoring the growth of individual colonies over time was possible (Figure 1). Microcolonies were first detected at day 4–5 (with a 5X objective) and presumptively identified as microcolonies of *M. tuberculosis* before day 7.

This system used is very versatile, as not only colony growth *per se* can be measured, but also the effect of additives or changes in culture conditions on pre-formed microcolonies can be automatically determined after baseline calibration and colony identification on non-selective media (Figure 3). When a growing culture on the PAO support was moved to selective media, a change in response between susceptible and resistant colonies was detectable in less than two generation times (Figure 3A,C). Importantly, the effect of a change in media was most apparent when plotting the rate of change in individual colony size (Figure 3B,D) as opposed to average colony size adding further support to the value of monitoring colonies individually. Although a difference in growth could also be detected based on the average colony size, the time needed to differentiate between susceptibility and resistance was longer using this strategy than when individual colony growth rate was used. A further advantage of monitoring individual colonies is that when mixtures of strains with different susceptibilities are present the effect on a subpopulation of the colonies can still be readily and rapidly be identified (Figure 4). Thus, heterogeneity of microcolony phenotypes in a culture can be identified.

In this study, the distribution of colony sizes at each time point was in the range of 3–4 doubling times. Certainly a proportion of this variation is due to some CFUs consisting of multiple cells, although efforts to minimise, and quantify, this effect were undertaken by inoculating 5 µm filtered suspensions, and examining at high magnification (Figure 2). Variation in colony size is also partly due to neighbouring microcolonies merging at an early stage and subsequently being detected as a single colony, particularly when the inoculation density is high. In spite of this we believe that a large part of the variation remains biological, even though all the cells used in this study were all derived from log-phase cultures; i.e. there is detectable variation in the lag period between individual colonies. Therefore this method may have potential to investigate the distribution in lag times of different cell populations [13].

**Figure 5:**
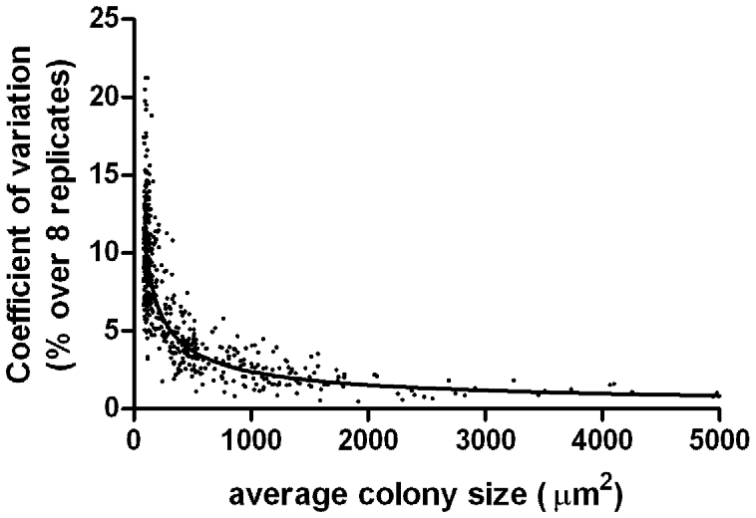
Coefficient of variation in object size vs. average object size over 8 separate measurements of approx. 500 objects.

The use of a relatively low magnification makes precise measurement of the size of the colonies at the early time points impractical. Small differences in focussing, the background structure of the support material and the large perimeter/area ratio of small colonies all contribute to errors in size measurements. In addition, the average colony size at the early time points (up to approx. day 5) is likely to be biased towards a selection of the larger colonies, because only colonies above the visual and analytical threshold (50 pixels) were included. As shown in Figure 5, the variation coefficient of the colony size measurements strongly decreases with colony size (to less than 5% above 168 pixels). We believe simplifying the analysis by resolving only slightly larger microcolonies outweighs the benefit of resolving single cells, where reliable automated analysis will be highly complex and probably impractical.

Our method, although performed only on cultured material to date, has the potential to detect the presence of *M. tuberculosis* at least as rapidly as other rapid TB culture methods, such as TLA (4–24 days) and MODS (4–31 days) automated liquid culture (6–24 days) [4], [9], [14].

A critical difference with the method described here is the approach for susceptibility testing. Direct susceptibility testing (DST) in MODS is performed by parallel inoculation of a sample in non-selective and selective media and this strategy can also be applied to liquid culture, but in reality sequential culturing is often performed for practical and cost reasons. Although the time gain of the DST is advantageous it leads to an expansion in the number of cultures, as all TB samples negative and positive are inoculated multiple times. Our method solves this problem as susceptibility testing is only performed on culture positive samples and, as susceptibility testing is preformed on established colonies, the time loss of subculturing is reduced.

As our novel approach for microcolony monitoring is particularly suited for further automation, we believe it has potential for use in both microbiological research and TB diagnostics. Nonetheless, obviously considerable research and development will be required to develop equipment and protocols for diagnostic purposes.

The versatility of the method demonstrated here for monitoring growth of colonies and the possibility to continue measurements after changing the culture conditions has many potential applications; differentiation of antibiotics with bactericidal or bacteriostatic activity after re-incubation on non-selective medium. The interaction between antibiotics, or detection of MDR strains studied by sequential incubations on different selective media. Additionally, approaches can be envisaged using solid porous supports subdivided into culture wells [11], for example different conditions tested in separate microcompartments or barriers created to prevent the overgrowth of rapidly growing or motile species. In conclusion, we believe the dramatic improvement in the performance and reduction of the cost of CCD imaging devices along with the rapid ongoing development of elegant and cost effective automated microscopy systems will make strategies such as those presented here, specifically designed to simplify the automated interpretation of microscopic images, increasingly important [7].

## Materials and Methods

### Culture

Tuberculosis strains used were *M. tuberculosis* H37Ra and HR1 (a spontaneous *in vitro* RIF resistant mutant derived from H37Ra, with rpoB H526Y mutation). *Mycobacterium tuberculosis* was routinely cultured in Middlebrook (MB)7H9 (Difco, BD, Sparks, MD, USA) medium supplemented with OADC (BBL, BD) in a shaker at 150 rpm and 37°C. For growth on solid medium, bacteria were plated on MB7H11(Difco, BD) agar supplemented with OADC. For growth measurements on selective media, MB7H11 containing 8 mg/L rifampicin was used.

### Culture on porous aluminium oxide (PAO)

Mycobacterial cultures were vortexed and centrifuged for 1 min at 100×g to remove clumps. Supernatants were filtered through a 5 µm filter (Whatman FP30/5.0CN-S, Kent, UK) to obtain a single cell suspension and diluted in MB7H9 medium to the desired cell density.

Strips of 1.5 cm×0.8 cm of porous aluminium oxide (PAO, trade name Anopore, Whatman, Kent, UK) were sterilized by autoclaving or submerging in 100% ethanol and dried strips were placed on MB7H11 agar.

Inoculation was performed by pipetting 5 µl suspension onto the surface and carefully spreading the liquid over the strip. Incubations were performed at 37°C.

To determine the percentage of single cells in the suspensions, auramine staining (using the FluoRAL kit; Réactifs RAL, Martillac, France) was also performed on aliquots of the suspensions smeared on slides.

### Microcolony growth microscopy

To investigate the feasibility of microscopic analysis of growing colonies of mycobacteria, cells were inoculated on PAO strips on agar, which were analysed for growth at regular intervals by low-power microscopy. In general 5 µl of a 1×10^6^ cells/ml suspension were inoculated onto each PAO strip (approx. 5000 cells).

Cultures were repeatedly imaged with an automated microscope system (FluXXscan; CCM Centre for Concepts in Mechatronics, Nuenen, The Netherlands) directly after inoculation (control; day 0) and daily from day 3 to day 10. At each time point, images were captured of identical fields by entering XY coordinates in the FluXXscan, so that microcolonies could be identified and their growth rate measured.

Ten microscopic fields of 2.8 mm^2^ each were analysed per series and condition.

### Nile red staining

To determine the growth at early time points and to estimate the number of cells per colony, additional PAO strips inoculated with *M. tuberculosis* for 0, 1, 2, 3 and 7 days were analysed using high power microscopy.

PAO strips containing bacteria were heat fixed (1 h, 60°C). Microscopic slides were prepared by spreading 1 ml of 1% molten agarose +0.5 µg/ml of the lipid probe Nile Red. After the agar was set, the heat-fixed PAO strips were placed on the agar for >1 h to allow the bacteria to incorporate the fluorescent probe. Slides were observed with 20 X or 40 X dry objectives.

### Drug susceptibility testing

To evaluate drug susceptibility testing by microcolony growth rate measurement, PAO strips on agar were inoculated as above with pairs of drug susceptible and resistant bacteria, respectively *M. tuberculosis* H37Ra and HR1 and incubated.

Once microcolonies were detected (approx. day 5), and growth was confirmed at a subsequent time point, PAO strips were carefully taken on day 7 from the non selective agar and transferred to agar containing the selective agent (or new non-selective agar as a control), after which growth measurements continued.

### Image analysis

Microcolony microscopy was performed using an automated microscopy system (FluXXscan) using bright field microscopy with coaxial illumination and 5X objective. Images were recorded using a monochrome CCD camera (XCL-X700, SONY, Park Ridge, NJ, USA; field size 1,024×768 pixels) and saved as TIF files.

For every experimental series 10 microscopic fields were photographed. Each microscopic field (2.8 mm^2^) was repeatedly imaged at day 0 and daily between days 3 and 10 after inoculation.

Photo analysis was performed using the open source Image J package (Rasband WS, 1997–2009 http://rsbweb.nih.gov/ij/).

#### Digital processing of images for analysis

Each series of images was loaded into ImageJ. Macros were written to perform the following analysis steps: A median filter (2 pixels) was applied followed by a background reduction step (“nonuniform background removal”; Quammen C, 2007 http://www.cs.unc.edu/~cquammen/imagej/nonuniform_background_removal.html). Then each set of images representing one of the 10 positions at all time points were stacked and aligned/registered and saved as JPEG files (“Register ROI”; Abramoff M, 1999–2006, http://bij.isi.uu.nl/). These images were made binary using the “RenyiEntropy” auto-thresholding tool (ImageJ). The sizes, circularity, and XY coordinates of all particles with a minimal size threshold of 50 pixels and circularity above 0.6 (Analyse particles, ImageJ) were saved to a data file (text file).

#### Data analysis

A web application was built in php-scripting language (Lerdorf R, 1994–2010, www.php.net) combined with a MySQL database (Axmark D, Larsson A and Widemius M, 1995–2009, www.mysql.com) in which all text files (resulting from ImageJ analysis) and registered images were loaded for further analysis.

In this application, data from all text files from each field at all time points were combined. Particles with similar XY coordinates (XY distance was less than the radius of the smaller of the two particles) present in at least two sequential time points were classified as a single object. All other particles were eliminated from further analysis. In this way individual colonies could be tracked over multiple time points. Composite registered images were made available to visually confirm similarity between the positions of the colonies. The resulting data from each series were exported to MS Excel (Microsoft, Seattle, USA) for analysis.

Based on the measured properties of growing colonies of the targeted bacterial species we defined criteria that particles must fulfill to be considered a colony, all other particles were excluded from further analysis. For all experiments, growth conditions up to day 7 were identical thus we base colony identifications on data up to day 7 and effects of any subsequent manipulations were followed on flagged colonies only. The primary criteria were; all particles detected in images at day 0 were discarded as background; secondly objects decreasing in size between the first day of detection and day 7 were discarded. Additionally we filtered on colony growth rate: Growth rate was recorded as % increase in colony area over 24 h. The distribution of growth rates was plotted for every series and time point. In general, these distribution graphs showed two peaks, representing two different “growth rates”, for every time point in each series; a growth rate around 0 (non-growing particles) and the real *M. tuberculosis* growth rate which was (depending on strain/condition) approximately a doubling in colony size per 24 h. Thus, our final criterion was that the area of the colony should increase between 25 and 200% in a 24 hour period prior to day 7.

### Statistical analysis

Data from growth curves were tested using unpaired T-tests between days 7 and 10. The effect of change in colony growth rate after change to selective media was tested using paired T-tests.
